# Gut microbiome is associated with metabolic syndrome accompanied by elevated gamma-glutamyl transpeptidase in men

**DOI:** 10.3389/fcimb.2022.946757

**Published:** 2022-07-29

**Authors:** Shifeng Sheng, Su Yan, Jingfeng Chen, Yuheng Zhang, Youxiang Wang, Qian Qin, Weikang Li, Tiantian Li, Meng Huang, Suying Ding, Lin Tang

**Affiliations:** ^1^ Health Management Center, The First Affiliated Hospital of Zhengzhou University, Zhengzhou, China; ^2^ College of Public Health, Zhengzhou University, Zhengzhou, China; ^3^ Department of Nephropathy, The First Affiliated Hospital of Zhengzhou University, Zhengzhou, China

**Keywords:** metabolic syndrome, glutamyl transpeptidase, gut microbiota, metagenomics, metabolic pathway, polyamine, endogenous alcohol

## Abstract

It is predicted that by 2035, metabolic syndrome (MS) will be found in nearly more than half of our adult population, seriously affecting the health of our body. MS is usually accompanied by the occurrence of abnormal liver enzymes, such as elevated gamma-glutamyl transpeptidase (GGT). More and more studies have shown that the gut microbiota is involved in MS; however, the correlation between gut microbiota and MS with elevated GGT has not been studied comprehensively. Especially, there are few reports about its role in the physical examination of the population of men with MS and elevated GGT. By using the whole-genome shotgun sequencing technology, we conducted a genome-wide association study of the gut microbiome in 66 participants diagnosed as having MS accompanied by high levels of GGT (case group) and 66 participants with only MS and normal GGT level (control group). We found that the number of gut microbial species was reduced in participants in the case group compared to that of the control group. The overall microbial composition between the two groups is of significant difference. The gut microbiota in the case group is characterized by increased levels of “harmful bacteria” such as *Megamonas hypermegale*, *Megamonas funiformis*, *Megamonas* unclassified, *Klebsiella pneumoniae*, and *Fusobacterium mortiferum* and decreased levels of “beneficial bacteria” such as *Faecalibacterium prausnitzii*, *Eubacterium eligens*, *Bifidobacterium longum*, *Bifidobacterium pseudocatenulatum*, *Bacteroides dorei*, and *Alistipes putredinis*. Moreover, the pathways of POLYAMSYN-PWY, ARG+POLYAMINE-SYN, PWY-6305, and GOLPDLCAT-PWY were also increased in the case group, which may play a role in the elevation of GGT by producing amine, polyamine, putrescine, and endogenous alcohol. Taken together, there are apparent changes in the composition of the gut microbiome in men with MS and abnormal GGT levels, and it is high time to discover specific gut microbiome as a potential therapeutic target in that population. More in-depth studies of relevant mechanism could offer some new methods for the treatment of MS with elevated GGT.

## 1 Introduction

Metabolic syndrome (MS) is a complex group of metabolic diseases in the pathological state characterized by abdominal obesity, hypertension, insulin resistance, and hyperlipidemia, defined by the World Health Organization (WHO) (Saklayen, 2018). At present, the prevalence of MS is increasing all over the world, and it has become a major problem endangering public health and seriously affecting the quality of life. The International Diabetes Federation (IDF) has reported that approximately 25% of the total world population is suffering from MS (Saklayen, 2018). It is predicted that the prevalence of MS will increase to 53% by 2035 (Engin, 2017). MS is also a cause of lipid deposition in hepatocytes and is closely associated with hepatocyte necrosis and dysfunction. Studies have found that elevated gamma-glutamyl transpeptidase (GGT) is closely related to atherosclerosis, cardiovascular disease, and impaired glucose tolerance (Franzini et al., 2013) and can predict the risk of diabetes, hypertension, MS, and cardiovascular disease independently (Onat et al., 2012; Yadav et al., 2017; Shiraishi et al., 2019). The possible reason is that GGT not only participated in the hydrolysis of extracellular glutathione but also is a marker of oxidative stress and subclinical inflammation (Alissa, 2018), both of which are considered as important mechanisms for atherosclerosis and the occurrence of MS. However, the current focus of the prevention and treatment of MS is just improvement of lifestyle and comprehensive control of various metabolic abnormal factors and lacks a specific treatment plan. Hence, it is high time to further study the mechanism of MS with elevated GGT and further explore effective treatment measures.

In recent years, with the quick development of high-throughput sequencing technology, the close association between intestinal microbiota and MS has gradually attracted our attention. Glycolipid metabolism disorders, oxidative stress, and inflammatory reactions in the process of MS can cause the alteration of intestinal microbiota, and the disturbances of intestinal microbiota can also accelerate the progression of MS. Studies have shown that the intestinal microbiota is an “energy metabolism organ” of our host (Ley et al., 2006; Sadik et al., 2010), and it can affect the metabolism of various nutrients such as sugar, fat, and protein and maintain the normal function of the intestinal mucosa (Anhe et al., 2015). The possible mechanisms that gut microbiota is involved in MS are as follows: firstly, it can regulate the immune system and balance the immune response of our body (Furusawa et al., 2013); secondly, it may mediate low-grade inflammation by producing lipopolysaccharide (LPS) (Org et al., 2017); last but not the least, it can digest and utilize substances that the host cannot use and promote the absorption of polysaccharides (Lindheim et al., 2017). At present, the treatment of MS lacks specific multitarget therapeutic drugs. Thus, improving metabolic disorders through different methods may be a new treatment method, such as the regulation of the gut microbiota. Yet, research on whether the increase of GGT in patients with MS is related to the intestinal microbiota is rare, especially in the male physical examination population. In this study, asymptomatic physical examination population was selected as the research object to observe the characteristics of the gut microbiota of MS patients with elevated GGT. It may provide new therapeutic targets and personalized prevention strategies for the clinical treatment of MS with abnormal GGT level.

## 2 Materials and methods

### 2.1 Study design

**Figure 1:**
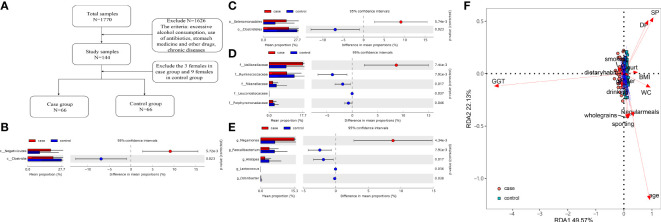
**(A)** Flow diagram. **(B–E)** Differences at the level of class, order, family, and genus level. **(F)** The redundancy analysis (RDA) showed the effect of the gammaglutamyl transpeptidase (GGT) level and individual attributes on microbiota.

A total of 1 770 subjects were examined in our study from January 2018 to May 2019, and the Ethics Committee from the First Affiliated Hospital of Zhengzhou University approved this study (Approval numbers: 2018-KY-56 and 2018-KY-90). Inclusion criteria are as follows: 1) age >18 years; 2) clinically diagnosed MS and elevated GGT level (the exact level of GGT is 50 U/L) according to relevant guidelines (Grundy et al., 2005; Hsieh et al., 2009). Exclusion criteria are as follows: 1) other factors that affect liver enzymes such as hepatitis B/C, autoimmune, and alcohol or drug-induced hepatitis (all of the subjects were prohibited from alcohol consumption at least 3 days before the exsanguination); 2) women in pregnancy or lactation; 3) subjects who were being treated with antibiotics, microbiota regulators, yogurt, probiotics, or proton pump inhibitors within the past 2 months; 4) subjects with diabetes, hypertension, coronary atherosclerotic heart disease, hyperthyroidism, hypothyroidism, Cushing syndrome, and so on. A total of 69 people and 75 participants were randomly enrolled in the case group (MS with elevated GGT level) and control group (MS with normal GGT level), respectively. Three women in the case group and nine women in the control group were excluded. The flow diagram is shown in **Figure 1A** and their profile is summarized in **Table 1**.

**Table 1:** The major characteristics and laboratory test results in male patients of the case and control groups.

Feature	Control (n = 66)	Case (n = 66)	*P*
Age	45.26 ± 8.11	43.47 ± 8.83	*0.23*
WC	94.93 ± 4.92	97.15 ± 8.31	*0.09*
SBP	138.70 ± 14.91	143.05 ± 13.02	*0.08*
DBP	88.47 ± 10.55	91.65 ± 10.29	*0.08*
BMI	28.47 ± 1.90	28.93 ± 2.78	*0.27*
WBC	6.34 ± 1.41	6.97 ± 1.35	0.01*
PLT	221.71 ± 45.67	240.3 ± 60.64	0.04*
NEUT	3.77 ± 1.02	4.08 ± 1.00	*0.08*
MON	0.37 ± 0.11	0.47 ± 0.12	<0.01**
BASO	0.03 ± 0.02	0.04 ± 0.02	0.016*
ALT	30.82 ± 13.02	46.89 ± 36.9	<0.01**
AST	21.58 ± 5.58	29.55 ± 17.21	<0.01**
GGT (U/L)	31.02 ± 9.66	96.00 ± 47.88	<0.01**
ALB	48.74 ± 2.46	48.94 ± 2.69	*0.645*
TBIL	12.16 ± 4.09	12.96 ± 6.65	*0.41*
Crea	75.21 ± 11.71	73.14 ± 11.05	*0.30*
SUA	376.67 ± 97.02	392.79 ± 76.31	*0.29*
TC	4.79 ± 0.87	5.19 ± 0.85	0.01*
TG	2.51 ± 1.09	3.33 ± 2.52	0.02*
HDL	1.13 ± 0.26	1.15 ± 0.26	*0.62*
LDL	2.99 ± 0.75	3.18 ± 0.78	*0.15*
FBG	6.46 ± 2.02	6.68 ± 2.02	*0.53*
MAFLD	NO 11; YES 55	NO 5; YES 61	*0.11*
Regular meals	NO 15; YES 51	NO 25; YES 41	*0.058*
Dietary habit	mix19; meatarian 44; vegetarian 3	mix 15; meatarian 36; vegetarian 15	0.01*
Wholegrains	NO 21; YES 45	NO 33; YES 33	0.034*
Yogurt	NO 36; YES 30	NO 41; YES 25	*0.377*
Smoking	NO 38; YES 28	NO 28; YES 38	*0.082*
Drinking	NO 20; YES 46	NO 19; YES 50	*0.434*
Sporting	not 19; rarely 24; frequently 23	not 21; rarely 28; frequently 17	*0.52*

WC, waist circumference; BMI, body mass index; DBP, diastolic blood pressure; SBP, systolic blood pressure; FBG, fasting blood glucose; AST, aspartate aminotransferase; ALT, alanine aminotransferase; GGT, gamma-glutamyl transpeptidase; ALB, albumin; TBIL, total bilirubin; SUA, serum uric acid; Crea, serum creatinine; TC, total cholesterol; TG, triglyceride; LDL, low-density lipoprotein; HDL, high-density lipoprotein; WBC, white blood cell; NEUT, absolute value of neutrophil; MO, monocyte absolute value; BASO, absolute basophil; PLT, platelet; MAFLD, metabolic-associated fatty liver disease. ^*^ *P*< 0.05 and ^**^ *P*< 0.01.

### 2.2 Measurement data and laboratory tests

The height, weight, waist circumference (Computerized body scale, SK-X80), and blood pressure (OMRON Medical automatic electronic blood pressure monitor, HBP-9021) were measured by trained staff, and body mass index (BMI) (kg/m^2^) was calculated as weight/height^2^. All blood samples were examined by UniCel DxI 800 Immunoassay System from Beckman Coulter: alanine aminotransferase (ALT), aspartate aminotransferase (AST), gamma-glutamyl transpeptidase (GGT), fasting blood glucose (FBG), total bilirubin (TBIL), low-density lipoprotein (LDL), high-density lipoprotein (HDL), triglyceride (TG), total cholesterol (TC), albumin (ALB), serum creatinine (Crea), serum uric acid (SUA), white blood cell (WBC), monocyte absolute value (MO), absolute value of neutrophil (NEUT), absolute basophil (BASO), and platelet (PLT).

Meanwhile, a Toshiba Color Doppler Ultrasound System (APLI0500 TUS-A500, Japan) was applied to scan the abdomen by a linear array probe with a frequency of 5–12 MHz. Two mid-level or above sonographers jointly cooperate with each other to observe the changes of the echo in the near and far fields of the liver and structure of the intrahepatic ducts, then determine whether the liver cells have steatosis, that is, metabolic-associated fatty liver disease (MAFLD).

### 2.3 Stool sample collection

About 1 g of feces specimens were obtained from recruited participants before 10 o’clock on the day, and the microbial sample preservation tubes were immediately stored in −20°C and then transferred to -80°C refrigerator for frozen preservation within 30 min.

### 2.4 Microbiota composition and function profiling

According to the manufacturer’s instructions, we extracted DNA from 132 stool samples using a MagPure Stool DNA KF kit B (Magen, China). Qubit Fluorometer was used for the quantification of DNA, prepped with the Qubit dsDNA BR Assay kit (Invitrogen, USA). All genomic DNA was broken to form random fragments by ultrasound and be selected. The selected fragments were then amplified and purified to obtain probe-anchored synthesis technology (cPAS) (MGI2000, MGI, Shenzhen, China). The final library sequenced on BGISEQ-500 platform (BGI-Shenzhen, China) was formed after formatting and identified by quality control. Hybrid sequences such as food genome sequence, low-quality sequence, and human genome sequence were eliminated by performing the sequencing data as the qualitative control. MetaPhlAn2 with default settings was applied to classify and annotate the metagenome of the sequencing library and generate the standard gut microbial profiling species at all levels such as bacteria, archaea, viruses, and eukaryotes (Truong et al., 2015). The NCBI.nlm.nih.gov database (National Center for Biotechnology Information, 2014 Edition) and HUMAnN2 (the HMP Unified Metabolic Analysis Network 2) were used to annotate the non-redundant gene set and the functional genes into Kyoto Encyclopedia of Genes and Genomes (KEGG) metabolic pathway and generated the composition of the metabolic pathway (Fang et al., 2018; Li et al., 2021).

### 2.5 Statistical analysis

R (version 4.0.5) was used for the statistical analyses. Laboratory test, demography, bacterial species, and pathways were analyzed by standardized statistical tests. Categorical variables were represented by counts, and chi-square tests were used for differential analyses. Continuous variables were expressed as means ± standard deviation ( $\bar{x}$ ± s). Normality tests and homogeneity tests were used for the analysis of between-group differences, and *P* ≥ 0.05 was selected as the normal and homogeneity variances. The Student’s t-test or Mann–Whitney test was used for analyzing the normal and homogeneous results, respectively, and *P*< 0.05 was regarded as statistically significant. The permutational multivariate analysis of variance (PERMANOVA) and redundancy analysis (RDA) were performed by us to confirm whether elevated GGT level was the most important influencing factor between the two groups. The package of “ADE4” in R program was applied to perform the principal coordinate analysis (PCoA), and the package of “vegan” was used to obtain Shannon, Gini, Hellinger, Jensen–Shannon divergence (JSD), and Bray indexes for each sample. STAMP (version 2.1.3) was uses to analyze the difference in the microbiome at the phylum through species levels and pathways. Welch’s t-test and multiple test correction using the Benjamini–Hochberg false discovery rate (FDR) were applied to calculate the difference between two groups. The species with low occurrence rates and expression levels (positivity rates<10%) were removed. The correlations between gut microbiota and covariates were analyzed by Spearman correlation method and the “corrplot” package was used for visualization.

## 3 Results

### 3.1 Clinical characteristics

There were 66 MS patients with elevated GGT (case group) and 66 MS patients with normal GGT (control group) in our cross-sectional cohort study. The level of GGT accompanied by ALT, AST, WBC, MON, BASO, PLT, TC, TG, Dietary habit, and Wholegrains in the case group were significantly higher than those in the control group. Moreover, the incidence of MAFLD in the case and control groups is 92.42% and 83.33%, respectively (**Table 1**).

### 3.2 Analysis of factors affecting the gut microbiota in the species level

First, we use multivariate variance (PERMANOVA) to analyze the response factor to gut microbiota changes in the species level and use a distance matrix (Bray–Curtis distance) to decompose the total variance. The interpretation degree of different grouping factors on sample differences was analyzed, and permutation tests were used to analyze whether different response variables had a significant impact on bacterial community structure. Specifically, we analyzed the basic information of the included population (i.e., GGT level, Regularmeals, Dietary habit, Wholegrains, Yogurt, Smoking, Drinking, Sporting, Age, WC, BMI, SBP, DBP) *via* PERMANOVA. Both univariate and multivariate analyses showed that the GGT level had the greatest effect on participants’ gut microbiota structure (*P*< 0.05, **Table 2**).

**Table 2:** The influence of the basic attributes of the participants on the gut microbiome in the species level.

Phenotype	Single factor			Multifactor		
	F. Model	Variation (R^2^)	Pr (>F)	F. Model	Variation (R^2^)	Pr (>F)
GGT	2.807	0.021	0.004	2.815	0.021	0.002
Regular meals	0.989	0.008	0.402	1.186	0.009	0.261
Dietary habit	0.523	0.004	0.933	0.457	0.003	0.967
Wholegrains	0.711	0.005	0.74	0.874	0.007	0.534
Yogurt	0.933	0.007	0.471	1.04	0.008	0.357
Smoking	1.025	0.008	0.393	0.623	0.005	0.86
Drinking	0.881	0.007	0.583	0.708	0.005	0.75
Sporting	0.739	0.006	0.704	0.896	0.007	0.511
Age	1.382	0.011	0.172	1.405	0.011	0.156
WC	1.083	0.008	0.337	1.25	0.009	0.228
SP	1.175	0.009	0.282	1.171	0.009	0.248
DP	1.081	0.008	0.338	0.955	0.007	0.483
BMI	1.04	0.008	0.378	1.812	0.014	0.048

WC, waist circumference; BMI, body mass index; DBP, diastolic blood preassure; SBP, systolic blood preasure; GGT, gamma-glutanyl transpeptidase.

### 3.2 Differences in the microbiota at all levels

Together, 16 phyla, 25 classes, 37 orders, 76 families, and 170 genera were found. The differences in the microbiota were calculated by STAMP from the level of phylum to species. Benjamini–Hochberg FDR was used for the correction of Welch’s t-test and multiple tests. In total, 2 classes, 2 orders, 5 families, and 5 genera (**Figures 1B–E**) showed a difference between the two groups. We also constructed the RDA diagram to show the relevance between the microbiome and subjects’ individual attributes and cohort (**Figure 1F**). The RDA was applied to reflect the samples and response factors on the same two-dimensional ranking map from which the relationship between the sample distribution and the basic characteristics of the host can be seen intuitively.

### 3.4 Community diversity in the case and control groups

**Figure 2:**
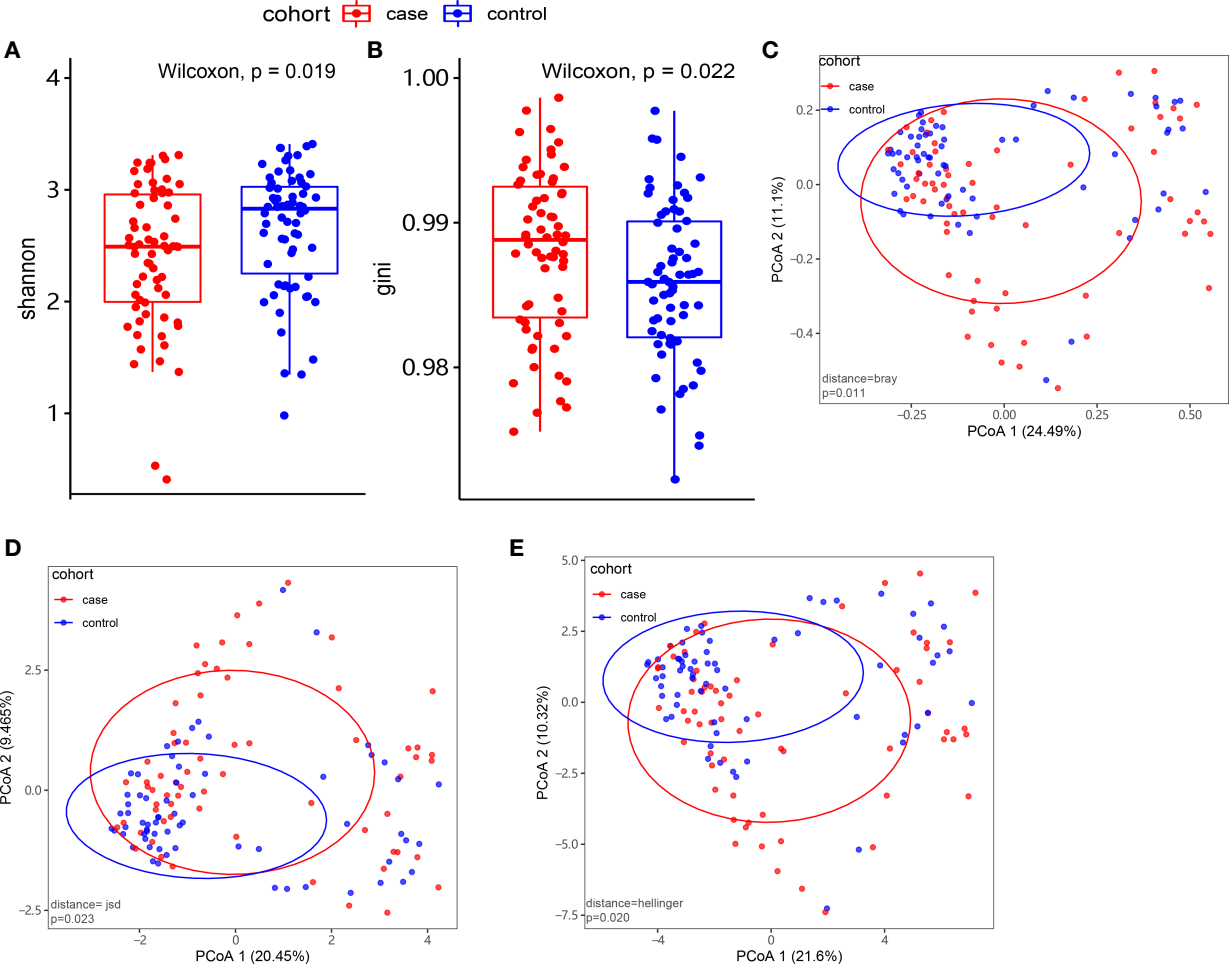
Microbiome composition and diversity. **(A, B)** Alpha diversity by Shannon and Gini indexes between the case group (N = 66) and control group (N = 66). **(C–E)** Beta diversity by Bray, Hellinger, and Jensen–Shannon divergence (JSD) indexes between the two groups.

At the species level, we applied alpha and beta diversity to evaluate the community diversity. In **Figures 2A, B**, the alpha diversity by Shannon index and Gini index showed a significant difference between the case and control groups (*P*< 0.05). In the species-level beta diversity, Bray distance, Hellinger distance, and Jensen–Shannon divergence (JSD) distance were calculated, and all of them showed significant differences (*P*< 0.05, **Figures 2C–E**).

### 3.5 The gut microbiome characteristics in different groups and the association with clinical index

#### 3.5.1 The gut microbiome characteristics in different groups

**Figure 3:**
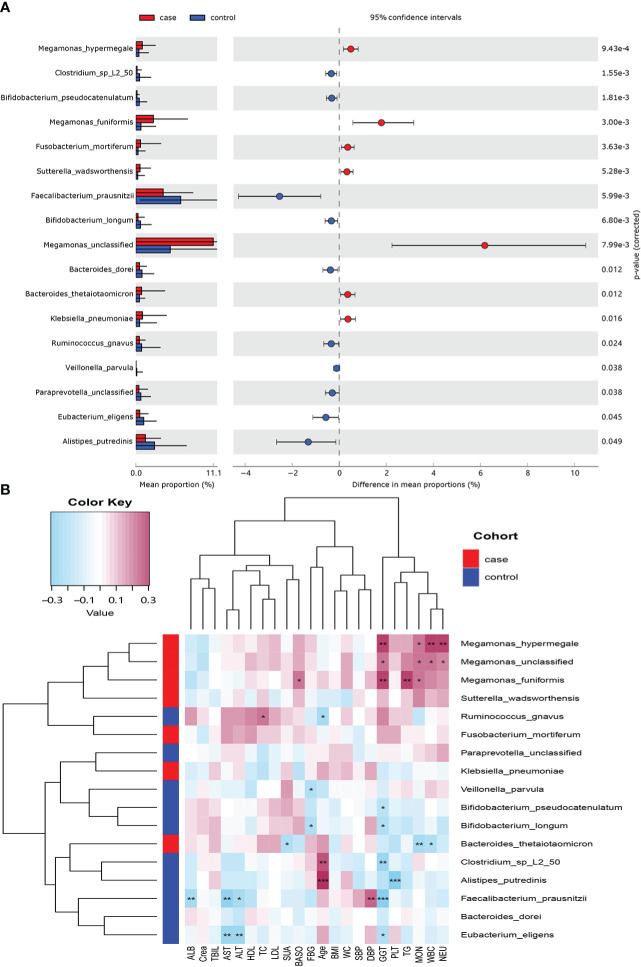
Microbiome difference between the case and control groups and the correlation with clinical index (*P-value*_corrected<_0.05). **(A)** The relative abundance of bacterial species with significant difference between the two groups. **(B)** Correlation matrix of the bacterial species and clinical index. Blue cell color represented a negative correlation; red cell color represented a positive correlation. ^*^ *P*< 0.05, ^**^ *P*< 0.01, and ^***^ *P*< 0.001.

Seventeen species showed a significant difference between the two groups at the species level (*P*< 0.05; **Figure 3A**). A total of 7 species including *Megamonas hypermegale*, *Megamonas funiformis*, *Megamonas* unclassified, *Fusobacterium mortiferum*, *Sutterella wadsworthensis*, *Bacteroides thetaiotaomicron*, and *Klebsiella pneumoniae* were enriched in the case group; 10 species including *Bifidobacterium longum*, *Bifidobacterium pseudocatenulatum*, *Faecalibacterium prausnitzii*, *Bacteroides dorei*, *Alistipes putredinis*, *Clostridium sp_L2_50*, *Ruminococcus gnavus*, *Paraprevotella* unclassified, *Eubacterium eligens*, and *Veillonella parvula* were enriched in the control group.

#### 3.5.2 Association between the gut microbiota and clinical index

We used Spearman’s correlation analysis to explore the correlation between the abundance of species and participants’ features. Except ALB, TBIL, and Crea, most of the gut microbiota enriched in the case group had positive relationships with these clinical indexes, such as *M. hypermegale*, *M. funiformis*, *Megamonas* unclassified, *S. wadsworthensis*, and *F. mortiferum*; especially *M. hypermegale*, *M. funiformis*, and *Megamonas* unclassified had a significantly positive correlation with the marker of inflammation (i.e., NEU, WBC, and MOC) and metabolic indicators (i.e., TG and GGT). While those microbiomes enriched in the control group had negative correlations with most clinical indexes, such as *B. longum*, *B. pseudocatenulatum*, *F. prausnitzii*, *B. dorei*, *A. putredinis*, *Clostridium sp_L2_50*, and *E. eligens*, *especially F. prausnitzii* and *E. eligens* had the most negative association with liver enzymes (GGT, ALT, and AST); *A. putredinis* was highly negatively correlated with PLT (**Figure 3B**).

### 3.6 Functional shifts in the microbiome characteristics in the case and control groups

#### 3.6.1 The functional shifts from the contrast of different subjects

**Figure 4:**
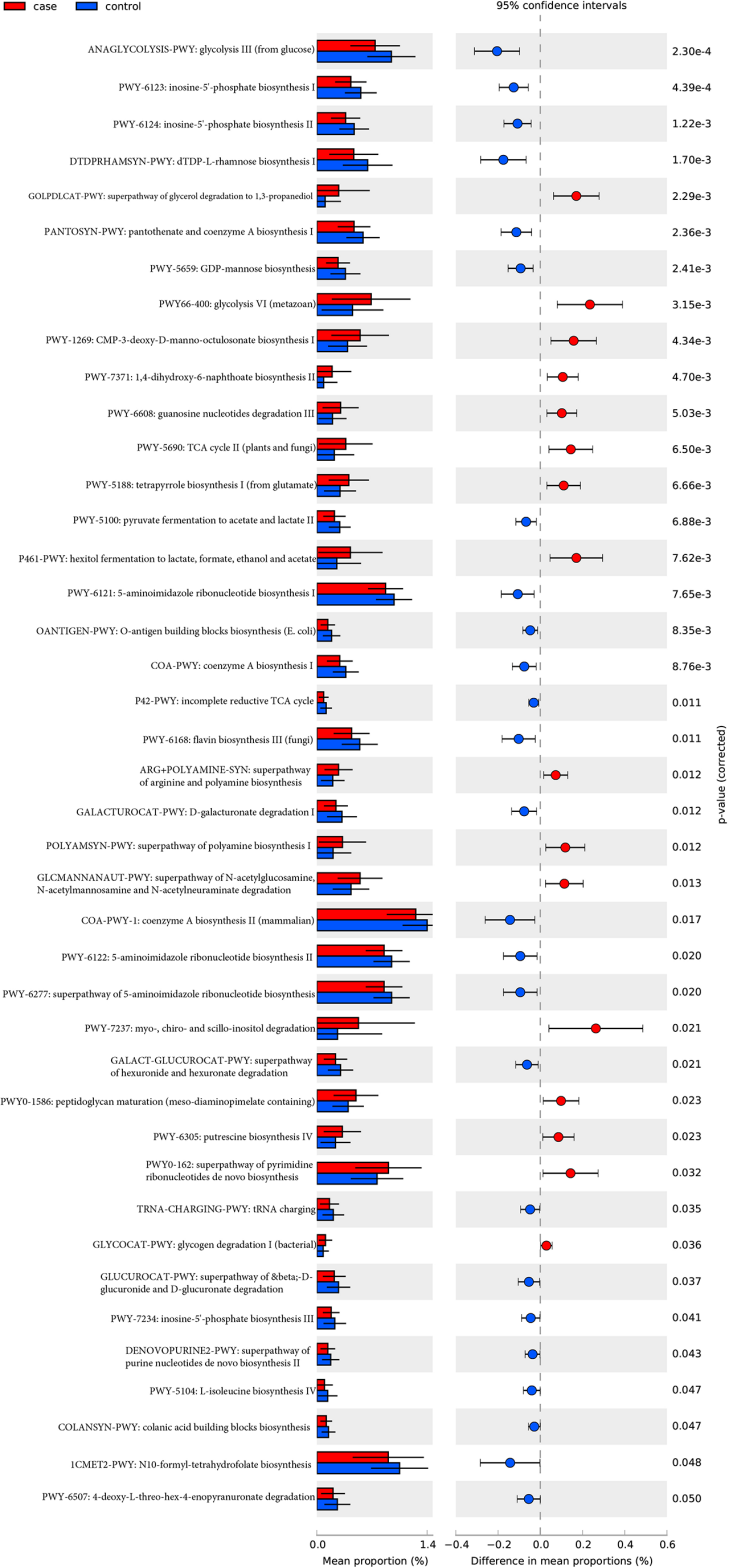
MetaCyc pathways with a significant difference in abundance between the case and control groups (*P*-value _corrected<_0.05).

A total of 494 microbial MetaCyc pathways were applied to construct the function profile. Forty-one pathways showed a significant difference between the two groups after removing the low occurrence rate pathways (positivity rates<10%), and 16 were enriched in the case group (**Figure 4**). Within these 16 pathways, three were for biosynthesis of amine and polyamine (ARG+POLYAMINE-SYN, POLYAMSYN-PWY, and PWY-6305) and one was for their degradation (GLCMANNANAUT-PWY); one was for fermentation of hexitols to lactic acid, formic acid, ethanol, and acetic acid (P461-PWY); one was for inositol degradation (PWY-7237); two pathways were in charge of producing the precursor metabolite and energy (PWY-5690 and GLYCOCAT-PWY); one was responsible for guanosine nucleotide degradation (PWY-6608); one was for glycerol degradation (GOLPDLCAT-PWY); and the others were for biosynthesis (PWY0-162, PWY-7371, PWY0-1586, and PWY-5188). For the pathways enriched in the control group, six were related to nucleoside and nucleotide biosynthesis process (DENOVOPURINE2-PWY, PWY-6121, PWY-6122, PWY-6123, PWY-6124, and PWY-7234); five were for biosynthesis of cofactor, carrier, and vitamin, which make large contributions to the Tricarboxylic acid cycle (TCA cycle) and redox reactions (1CMET2-PWY, COA-PWY-1, COA-PWY, PANTOSYN-PWY, and PWY-6168); seven were responsible for degradation of carbohydrates and generation of precursor metabolites and energy (ANAGLYCOLYSIS-PWY, GALACT-GLUCUROCAT-PWY, PWY-5100, GALACTUROCAT-PWY, GLUCUROCAT-PWY, P42-PWY, and PWY-6507); four were for carbohydrate biosynthesis (COLANSYN-PWY, DTDPRHAMSYN-PWY, OANTIGEN-PWY, and PWY-5659); one was for amino acid synthesis (PWY-5104); and one was for tRNA charging (TRNA-CHARGING-PWY).

#### 3.6.2 Relationship between functional shifts in the microbiome and the clinical index

**Figure 5:**
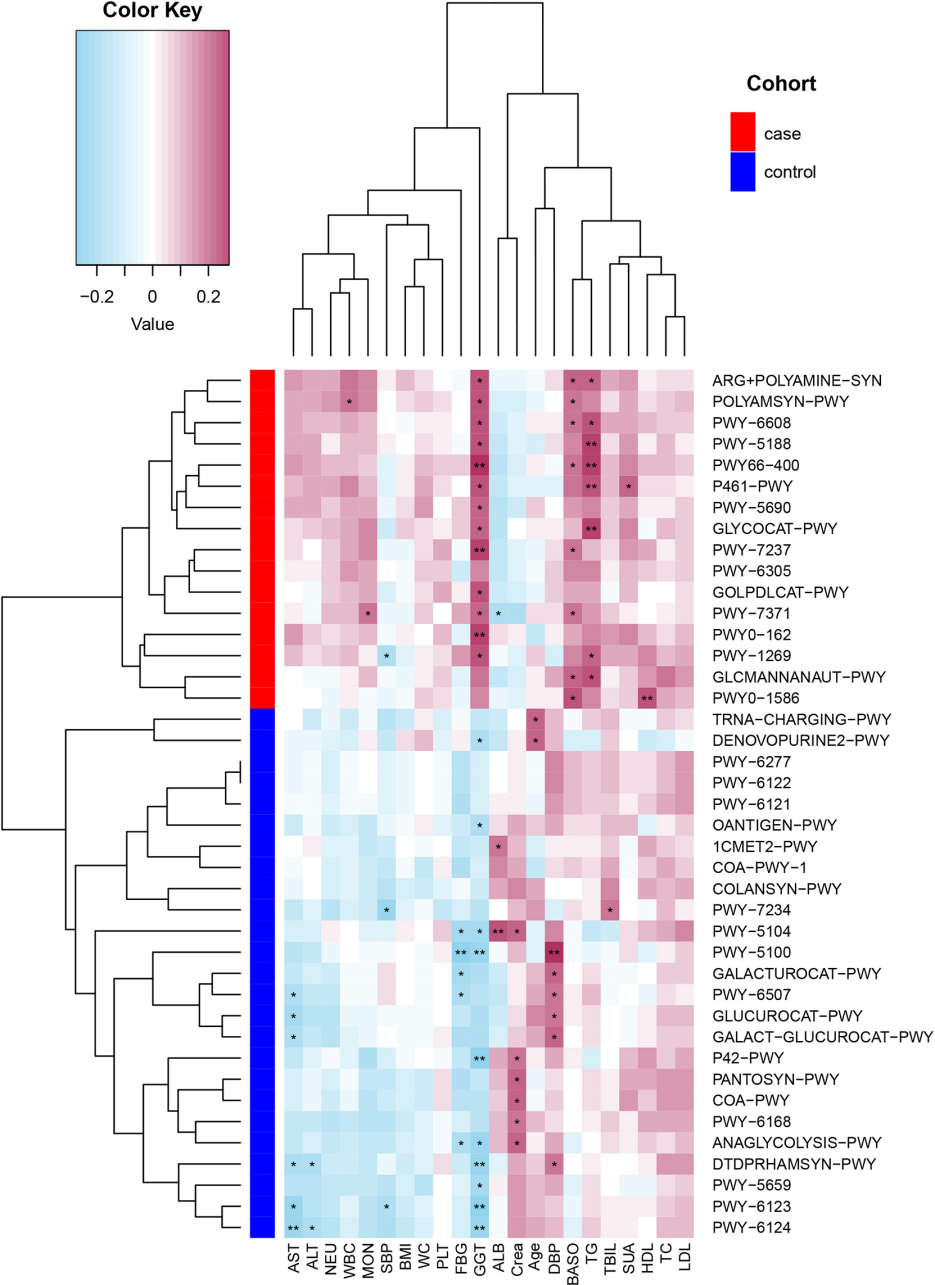
Spearman’s correlation matrix for the case and control group correlation pathways and clinical index. Blue cell color represented a negative correlation; red cell color represented a positive correlation. ^*^ *P*< 0.05 and ^**^ *P*< 0.01.

Spearman’s correlation analyses explored the relationships between functional shifts and clinical index, and then we constructed the heatmaps (*P*< 0.05, **Figure 5**). Except ALB and Crea, the most enriched pathways in the case group had positive associations with the clinical index, especially PWY-5188, PWY66-400, P461-PWY, and GLYCOCAT-PWY had a significant difference with GGT and TG, while the most enriched pathways in the control group had a negative relationship with FBG, liver function, and inflammation indexes.

#### 3.6.3 Relationship between functional shifts and microbiome characteristics

**Figure 6:**
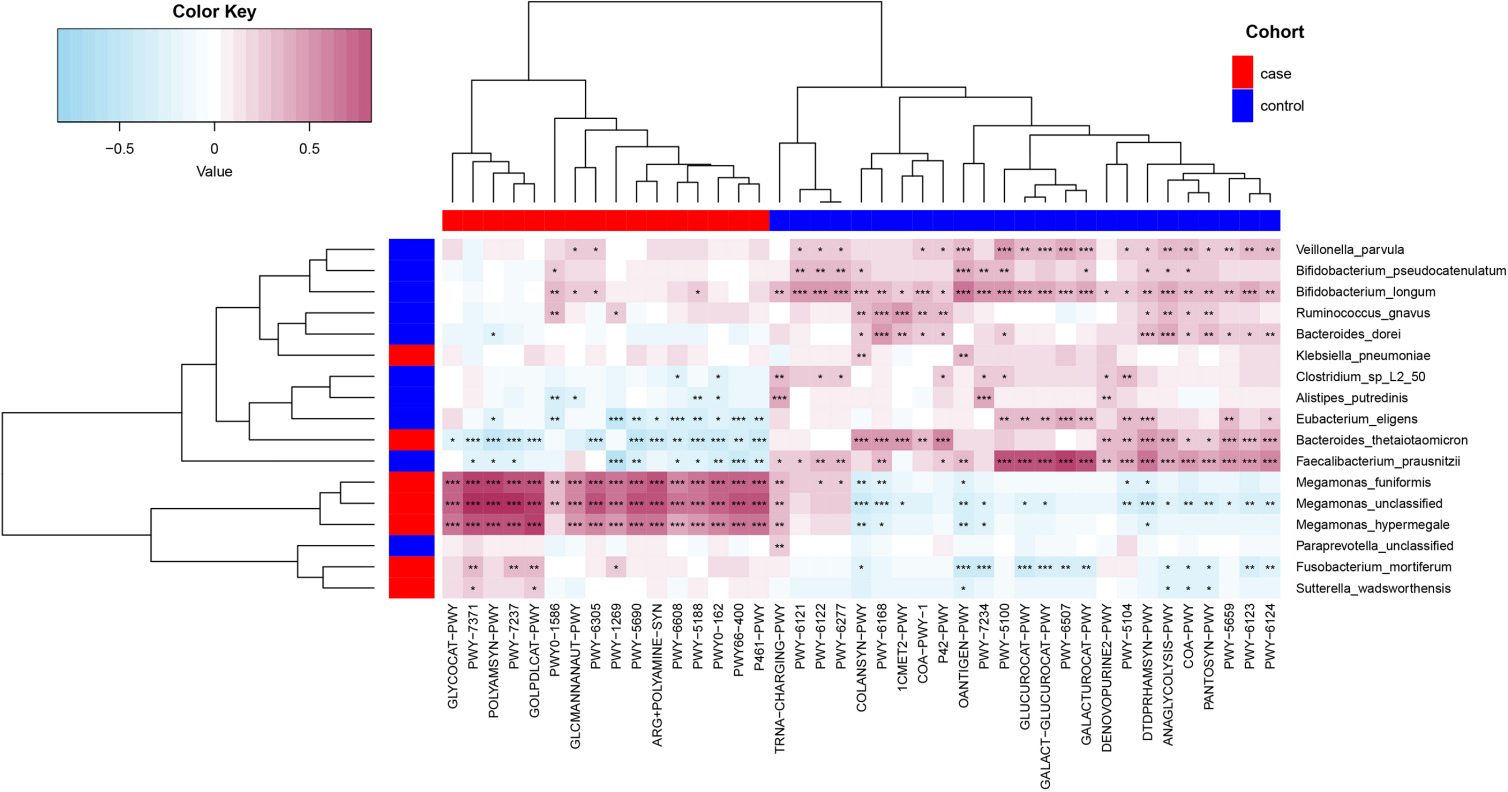
Spearman’s correlation matrix for functional shifts and microbiome characteristics in the case and control groups. Blue cell color represented a negative correlation; red cell color represented a positive correlation. ^*^ *P*< 0.05, ^**^ *P*< 0.01, and ^***^ *P*< 0.001.

Spearman’s correlation analyses were used to analyze the relationships between functional shifts and microbiome characteristics (*P*< 0.05), and then we constructed the heatmaps (**Figure 6**). Apparently, *Megamonas* species and *F. Mortiferum*, enriched in the case group, had significantly positive relationships with all pathways enriched in the case group (especially the pathways that were responsible for the biosynthesis of amine, polyamine, and putrescine, such as POLYAMSYN-PWY, ARG+POLYAMINE-SYN, and PWY-6305; the biosynthesis of 1,4-dihydroxy-6- naphthoate such as PWY-7371; and the degradation of glycerin and myo-, chiro-, and scillo-inositol, such as GOLPDLCAT-PWY and PWY-7237) and negative relationships with most pathways enriched in the control group. *F. prausnitzii*, *E. eligens*, *B. dorei*, *V. parvula*, *B. longum*, and *B. pseudocatenulatum*, enriched in the control group, had highly positive correlations with most pathways that were enriched in the control group, especially *F. prausnitzii* had the most significantly positive relationships with those pathways responsible for degradation of carbohydrates and generation of precursor metabolites and energy (GALACT-GLUCUROCAT-PWY, PWY-5100, GALACTUROCAT-PWY, GLUCUROCAT-PWY, PWY-6507, and ANAGLYCOLYSIS-PWY).

## 4 Discussion

A growing number of studies have shown that disruption of normal state gut microbiota is involved in the pathogenesis and development of multiple chronic low-grade inflammatory diseases, e.g., diabetes (Qin et al., 2012), obesity (Ley et al., 2006), hypertension (Manco et al., 2010), and liver cirrhosis (Qin et al., 2014), and the role of gut microbiome in MS has been gradually elucidated in recent years. Glycolipid metabolism disorders, oxidative stress, and inflammatory reactions in the process of MS can cause the alteration of gut microbiome, and the disturbance in the gut microbiota can also accelerate the progression of MS. At present, the treatment of MS still lacks specific multitarget therapeutic drugs. However, it may be a potential treatment method to regulate the gut microbiota and improve metabolic disorders through different methods. GGT is a direct marker of hepatic insulin resistance that can reflect the degree of hepatic fat deposition (Doi et al., 2007). Studies have shown that the increase in visceral fat is likely to be the main reason for the increase in liver enzyme levels (Pacifico et al., 2011). In addition, GGT is also involved in the oxidation stress response, which may mediate a chronic inflammatory process in MS. However, the research on whether the elevated GGT in patients with MS is related to the gut microbiota has been rarely reported, especially in the male physical examination population. According to our results, it is speculated that there may be some targeted gut microbiota biomarkers that can act as easy, safe, and non-invasive diagnostic tools, which could be used as a complement to traditional diagnostic methods for MS with abnormal GGT levels.

In our study, the level of GGT along with another two liver function indicators (ALT and AST), inflammation factors (WBC, MON, BASO, and PLT), and plasma lipid index (TC and TG) in the case group was obviously higher than that of the control group. Moreover, the level of SBP, DBP, and NEUT in the case group was slightly, but not significantly, higher than that of the control group. Increasing research has proven that the level of liver transaminase is related to the risk of MS (Chen et al., 2016; Kim and Han, 2018). A high level of primary GGT and the GGT value increase over time were both regarded as independent predictors of MS (Yadav et al., 2017). Just as Park et al. (2013) found that elevated liver enzymes were associated with hypertriglyceridemia, hypertension, etc., and there was an obvious dose–response correlation with the number of MS components. In addition, Oh et al. (2006) suggested that patients with metabolic abnormalities should pay much attention to the monitoring and analysis of liver enzyme levels even if they are not enough to diagnose MS. Even liver enzymes within the normal range are also related to the pathogenesis of MS (Steinvil et al., 2010; Zhang et al., 2014). Moreover, the incidence of MAFLD in the case group and control group is 92.42% and 83.33%, respectively. MAFLD is generally considered to be the liver manifestation of MS (Chen et al., 2020). The latest epidemiological data show that the prevalence of MAFLD in China rose from 25.4% in 2008–2010 to 32.3% in 2015–2018, of which 14%–25% MAFLD patients progressed to advanced fibrosis, and the prevalence of MAFLD in overweight and obese population was as high as 52.27% (Zhou et al., 2019), which is much lower than the result of this study, which may be due to region, diet, small population base, etc. Studies have shown that MAFLD and MS are mutually causal; these metabolic factors jointly promote diabetes, atherosclerosis, chronic kidney disease (CKD), coronary heart disease, liver cirrhosis, and the incidence of extrahepatic malignant tumors (Chalasani et al., 2018; Silva et al., 2018; Targher et al., 2020). Therefore, early prevention and intervention of the metabolism-related risks of MAFLD and MS are very important. Of course, the exact mechanism needs to be further studied urgently.

In this study, PERMANOVA was also used to analyze the basic information of the including population, and it was discovered that GGT was the most important influencing factor on the intestinal microbial structure. In terms of gut microbiome, not only the diversity of bacteria in the case group reduced obviously, but the microbial community was of obvious difference from that of the control group. Reduced diversity of microbiota was one of the main types of intestinal disease related to ecological imbalance (Requena et al., 2018) and was documented in many diseases, such as inflammatory bowel disease (IBD) (Frank et al., 2007), autoimmune hepatitis (Wei et al., 2020), and type 1 diabetes (Kostic et al., 2015). Researchers had shown that people with a reduction of microbial richness are more likely to develop chronic low-grade inflammation (Le Chatelier et al., 2013; Cotillard et al., 2013). Requena et al. (2018) once noticed that the microbiome diversity had close association with our health, and our result showed that the gut microbiota status has transformed significantly from MS to the development of MS with elevated GGT. We found that elevated GGT levels had significant effects on the gut microbiota, and the diversities of alpha and beta differed significantly. At the species level, compared with that of the control group, the relative abundance of *M. hypermegale*, *M. funiformis*, *Megamonas* unclassified, *F. mortiferum*, *S. wadsworthensis*, *B. Thetaiotaomicron*, and *K. pneumoniae* in the case group increased; *B. longum*, *B. pseudocatenulatum*, *F. prausnitzii*, *B. dorei*, *A. putredinis*, *Clostridium sp_L2_50*, *R. gnavus*, *Paraprevotella* unclassified, *E. eligens*, and *V. parvula* decreased. Interestingly, most of the gut microbes enriched in the case group (especially the *Megamonas*) showed significantly positive correlations with inflammatory markers (WBC, NEU, MON, and PLT), and the gut microbes in the control group had a negative correlation with them.

We found that the typical butyrate-producing gut microbiota such as *E. eligens* and *F. prausnitzii* were increased in the control group. Not only could they protect against inflammation by producing butyrate, a short-chain fatty acid (SCFA), but contribute to the gut integrity (Morrison and Preston, 2016). *In vitro* cell experiments have shown that *E. eligens* can strongly promote the generation of an anti-inflammatory cytokine, that is, interleukin (IL)-10 (Chung et al., 2017). *F. prausnitzii* is one of the most common bacteria in our intestinal microbiota (Lopez-Siles et al., 2017), and it was discovered to produce a microbial anti-inflammatory molecule (MAM), which can inhibit the pathway of nuclear factor (NF)-κB *in vitro* (Sokol et al., 2008; Quevrain et al., 2016; Breyner et al., 2017; Ganesan et al., 2018). This is consistent with our finding that *E. eligens* and *F. prausnitzii* were negatively correlated with inflammatory indicators. Moreover, *F. prausnitzii* had significant positive correlations with the pathways responsible for the generation of precursor metabolites and energy (GALACT-GLUCUROCAT-PWY, PWY-5100, GALACTUROCAT-PWY, GLUCUROCAT-PWY, PWY-6507, and ANAGLYCOLYSIS-PWY). *A. putredinis* decreases in both compensated and decompensated liver cirrhosis patients (Shao et al., 2018), and it was discovered to increase with the intakes of cruciferous vegetable in human body (Li et al., 2009). *B. dorei* has the effect of anti-influenza by enhancing the expression of earlier interferon, regulating the balance of pro- or anti-inflammatory cytokines, and reconstructing the composition of the gut microbiome (Song et al., 2021). *B. dorei* can also reduce the production of LPS, and treatment with live *B. dorei* may help prevent coronary artery disease (Yoshida et al., 2018) and treat obesity (Yoshida et al., 2021). Hence, *B. dorei* could be regarded as a new probiotic for the prevention and treatment of some clinical disease. Compositionally, bacteria belonging to *Bifidobacterium* genera were discovered to be enriched in or excited by the prebiotic in the lean microbiome, intimating the potential effect in leanness (Aguirre et al., 2016). Many studies have informed the anti-obesity effect of *Bifidobacterium* spp. (Ma et al., 2008; Yin et al., 2010; An et al., 2011), and some of them have been used as prebiotics in several diseases due to their immune-modulatory action. The bile acid excretion in feces was significantly increased in rats fed yogurt including *B. pseudocatenulatum* or *B. longum* (Al-Sheraji et al., 2012). *B. pseudocatenulatum* may ameliorate the gut homeostasis and impede the gut-derived complication in chronic liver disease (Moratalla et al., 2016). *B. longum LTBL16*, with a strong antioxidant activity, is a bacterial strain of potential probiotic isolated from Chinese healthy centenarians in Bama (Huang et al., 2020). *B. longum 51A* can protect mice against intestinal damage that was caused by irinotecan (Quintanilha et al., 2022). Taken together, the common feature of the above intestinal microbiota is that they are all generally considered “beneficial bacteria”. Most of them also showed the anti-inflammatory or anti-obesity effect in different studies, and the MS with elevated GGT is also a process of chronic low-grade inflammatory response, and there may be some link existing between these bacteria and this process. However, more further studies are required for exploring the exact relationship between them. This is an interesting direction worth exploring.

Numerous studies have confirmed the gut microbial community of obesity (Chiu et al., 2014; Maya-Lucas et al., 2019; Chen et al., 2020; Crovesy et al., 2020; Duan et al., 2021; Palmas et al., 2021), prediabetes (Zhang et al., 2013), primary aldosteronism (Liu et al., 2021), CKD, and hemodialysis patients (Lun et al., 2019), and dogs that have aggressive and phobic behavior (Mondo et al., 2020) exhibited an obvious increase in the *Megamonas* abundance, genera that are associated with not only inflammation (Hiippala et al., 2016; Ling et al., 2016; Lan et al., 2021) but also the metabolism of primary bile acids and abdominal pain in humans (Sakon et al., 2008; Yusof et al., 2017; Aleman et al., 2018). Moreover, members of *Megamonas* can produce acetic and propionic acids, which have been discovered to be substrates for the formation of lipogenesis and cholesterol in rodents (Conterno et al., 2011); more lipogenesis and cholesterol accumulation may lead to abnormal liver function. Especially the *Megamonas* species had highly positive correlations with the pathways responsible for the biosynthesis of amine, polyamine, and putrescine, such as POLYAMSYN-PWY, ARG+POLYAMINE-SYN, and PWY-6305 in this study. Studies have shown that the relative abundance of *Fusobacterium ulcerans* in hyperuricemia (Sheng et al., 2021) and post colorectal cancer surgery (Schmitt et al., 2021) was higher than that in the control group. As is known to all, *K. pneumoniae*, a Gram-negative pathogen bacterium of, is related to lots of opportunistic community-acquired infections. In anaerobic or aerobic conditions, *K. pneumoniae* can grow rapidly and produce high amounts of alcohol. High titers of 2,3-butanediol or 1,3-propanediol are the natural product of *K. pneumoniae*, and under micro-aerobic conditions, lactic acid is the main end product of fermentation, accompanied by some by-products such as ethanol, acetoin, acetic acids, formic acids, and succinic acids. In a previous Chinese cohort, researchers found that *K. pneumoniae* was associated with more than 60% of individuals with fatty liver disease (FLD), a precursor stage of liver cirrhosis and hepatocellular carcinoma. In addition, they proposed in some FLDs, the change in the gut microbiota can drive the condition by producing excess endogenous alcohol (Yuan et al., 2019). Moreover, they also showed that *K. pneumoniae* may lead to FLD *via* the production of endogenous ethanol, which is mediated by the pathway of 2,3-butanediol (Li et al., 2021). Consistent with previous research, our result showed that *K. pneumoniae* was enriched in the case group, and it has a positive correlation with GOLPDLCAT-PWY pathway, a super pathway for the degradation of glycerol to 1,3-propanediol. GGT is the most classic and sensitive indicator of alcoholic liver injury; accordingly, we can assume that *K. pneumoniae* may cause liver damage by producing high amounts of endogenous alcohol through the 1,3-propanediol pathway. There may exist some link between *K. pneumoniae* and abnormal liver function. Now, the gut microbiota has received much attention as a non-invasive biomarker for the prevention, diagnosis, and treatment of FLD (Sharpton et al., 2021). Fecal markers have been proposed for the diagnosis and prevention of colorectal and breast cancer subtypes (Yu et al., 2017; Banerjee et al., 2018). Hence, high-throughput sequencing of the above microbiota in feces may help in predicting the risk of abnormal liver enzymes in MS. This study provides a new direction on the diagnosis, prevention, and treatment of MS with elevated GGT.

This research selects the technology of whole-genome shotgun sequencing and chooses the population of asymptomatic physical examination as the study object to explore the disruption of gut microbiota in men with MS accompanied by abnormal GGT level, which are the highlights of the study. Our research also has limitations, such as this is a cross-sectional study, not detecting more inflammatory factors and bacterial metabolites, the gender in the two groups is male adult.

In summary, this research showed the alterations of structural and functional gut microbiome in men with MS accompanied by elevated GGT, which were characterized by increased levels of “harmful bacteria” such as *M. hypermegale*, *M. funiformis*, *Megamonas* unclassified, *K. pneumoniae*, and *F. mortiferum* and decreased levels of “beneficial bacteria” such as *F. prausnitzii*, *E. eligens*, *B. longum*, *B. pseudocatenulatum*, *B. dorei*, and *A. putredinis*. Moreover, the pathways of POLYAMSYN-PWY, ARG+POLYAMINE-SYN, PWY-6305, and GOLPDLCAT-PWY were also increased, which may play a role in the elevation of GGT by producing amine, polyamine, putrescine, and endogenous alcohol. Generally, the potential mechanisms underlying the gut microbiome and GGT accumulation link still need further exploration. The novel associations could provide novel direction for specific microbiome-targeted therapy.

## Data availability statement

The datasets presented in this study can be found in online repositories. The names of the repository/repositories and accession number(s) can be found below: https://db.cngb.org/, CNP0003031.

## Ethics statement

The study was approved by the ethics committee from the First Affiliated Hospital of Zhengzhou University (Number: 2018-KY-56 and 2018-KY-90). The patients/participants provided their written informed consent to participate in this study.

## Author contributions

Conceptualization, LT, SD, and SS. Data analysis, SY, JC, and YW. Samples collection, QQ, YZ, WL, TL, and MH. Original drafting, SS. Review and editing, LT and SD. Visualization, JC and SY. Project administration, LT and SD. All authors have read and agreed to summit the manuscript.

## Funding

This study was equally supported and funded by Henan Province Medical Science and Technology Research Plan (LHGJ20200311), Chinese National Science and Technology Major Project (2018ZX10305410), and Henan Province Key Scientific Research Projects of Universities (21A320035).

## Acknowledgments

The authors sincerely thank all participants or patients enrolled in this research. We also gratefully thank the clinicians and nurses from The First Affiliated Hospital of Zhengzhou University who assisted us with the questionnaire and sample collections, and TopEdit (www.topeditsci.com) for basic language editing of this manuscript.

## Conflict of interest

The authors declare that the research was conducted in the absence of any commercial or financial relationships that could be construed as a potential conflict of interest.

## Publisher’s note

All claims expressed in this article are solely those of the authors and do not necessarily represent those of their affiliated organizations, or those of the publisher, the editors and the reviewers. Any product that may be evaluated in this article, or claim that may be made by its manufacturer, is not guaranteed or endorsed by the publisher.

## Abbreviations

MS, metabolic syndrome; WC, waist circumference; BMI, body mass index; FBG, fasting plasma glucose; SBP, systolic blood pressure; DBP, diastolic blood pressure; ALT, alanine aminotransferase; AST, aspartate aminotransferase; GGT, gamma-glutamyl transpeptidase; TBIL, total bilirubin; ALB, albumin; TC, total cholesterol; TG, triglyceride; LDL, low-density lipoprotein; HDL, high-density lipoprotein; Crea, serum creatinine; SUA, serum uric acid; WBC, white blood cell; PLT, platelet; NEUT, absolute value of neutrophil; MON, monocyte absolute value; BASO, absolute basophil; SCFAs, short-chain fatty acids; LPS, lipopolysaccharide; MAFLD, metabolic-associated fatty liver disease; IDF, International Diabetes Federation; LPS, lipopolysaccharide; CKD, chronic kidney disease; MAM, microbial anti-inflammatory molecule.

## References

[B1] Aguirre M. Bussolo D. S. C. Venema K. (2016). The gut microbiota from lean and obese subjects contribute differently to the fermentation of arabinogalactan and inulin. PloS One 11 (7), e159236. doi: 10.1371/journal.pone.0159236 PMC4943740 27410967

[B2] Aleman J. O. Bokulich N. A. Swann J. R. Walker J. M. De Rosa J. C. Battaglia T.. (2018). Fecal microbiota and bile acid interactions with systemic and adipose tissue metabolism in diet-induced weight loss of obese postmenopausal women. J. Transl. Med. 16 (1), 244. doi: 10.1186/s12967-018-1619-z 30176893 PMC6122649

[B3] Alissa E. M. (2018). Relationship between serum gamma-glutamyltransferase activity and cardiometabolic risk factors in metabolic syndrome. J. Family Med. Prim. Care 7 (2), 430–434. doi: 10.4103/jfmpc.jfmpc_194_17 30090789 PMC6060918

[B4] Al-Sheraji S. H. Ismail A. Manap M. Y. Mustafa S. Yusof R. M. Hassan F. A. (2012). Hypocholesterolaemic effect of yoghurt containing bifidobacterium pseudocatenulatum G4 or bifidobacterium longum BB536. Food Chem. 135 (2), 356–361. doi: 10.1016/j.foodchem.2012.04.120 22868099

[B5] Anhe F. F. Roy D. Pilon G. Dudonne S. Matamoros S. Varin T. V.. (2015). A polyphenol-rich cranberry extract protects from diet-induced obesity, insulin resistance and intestinal inflammation in association with increased akkermansia spp. population in the gut microbiota of mice. Gut 64 (6), 872–883. doi: 10.1136/gutjnl-2014-307142 25080446

[B6] An H. M. Park S. Y. Lee D. K. Kim J. R. Cha M. K. Lee S. W.. (2011). Antiobesity and lipid-lowering effects of bifidobacterium spp. in high fat diet-induced obese rats. Lipids Health Dis. 10, 116. doi: 10.1186/1476-511X-10-116 21745411 PMC3146849

[B7] Banerjee S. Tian T. Wei Z. Shih N. Feldman M. D. Peck K. N.. (2018). Distinct microbial signatures associated with different breast cancer types. Front. Microbiol. 9. doi: 10.3389/fmicb.2018.00951 PMC5962706 29867857

[B8] Breyner N. M. Michon C. de Sousa C. S. Vilas B. P. Chain F. Azevedo V. A.. (2017). Microbial anti-inflammatory molecule (MAM) from faecalibacterium prausnitzii shows a protective effect on DNBS and DSS-induced colitis model in mice through inhibition of NF-kappaB pathway. Front. Microbiol. 8. doi: 10.3389/fmicb.2017.00114 PMC5285381 28203226

[B9] Chalasani N. Younossi Z. Lavine J. E. Charlton M. Cusi K. Rinella M.. (2018). The diagnosis and management of nonalcoholic fatty liver disease: Practice guidance from the American association for the study of liver diseases. Hepatology 67 (1), 328–357. doi: 10.1002/hep.29367 28714183

[B10] Chen S. Guo X. Yu S. Zhou Y. Li Z. Sun Y. (2016). Metabolic syndrome and serum liver enzymes in the general chinese population. Int. J. Environ. Res. Public Health 13 (2), 223. doi: 10.3390/ijerph13020223 26901209 PMC4772243

[B11] Chen X. Sun H. Jiang F. Shen Y. Li X. Hu X.. (2020). Alteration of the gut microbiota associated with childhood obesity by 16S rRNA gene sequencing. PeerJ 8, e8317. doi: 10.7717/peerj.8317 31976177 PMC6968493

[B12] Chen C. Zhu Z. Mao Y. Xu Y. Du J. Tang X.. (2020). HbA1c may contribute to the development of non-alcoholic fatty liver disease even at normal-range levels. Biosci. Rep. 40 (1), BSR20193996. doi: 10.1042/BSR20193996 31940026 PMC6997109

[B13] Chiu C. M. Huang W. C. Weng S. L. Tseng H. C. Liang C. Wang W. C.. (2014). Systematic analysis of the association between gut flora and obesity through high-throughput sequencing and bioinformatics approaches. BioMed. Res. Int. 2014, 906168. doi: 10.1155/2014/906168 25202708 PMC4150407

[B14] Chung W. Meijerink M. Zeuner B. Holck J. Louis P. Meyer A. S.. (2017). Prebiotic potential of pectin and pectic oligosaccharides to promote anti-inflammatory commensal bacteria in the human colon. FEMS Microbiol. Ecol. 93 (11), fix127. doi: 10.1093/femsec/fix127 29029078

[B15] Conterno L. Fava F. Viola R. Tuohy K. M. (2011). Obesity and the gut microbiota: Does up-regulating colonic fermentation protect against obesity and metabolic disease? Genes Nutr. 6 (3), 241–260. doi: 10.1007/s12263-011-0230-1 21559992 PMC3145060

[B16] Cotillard A. Kennedy S. P. Kong L. C. Prifti E. Pons N. Le Chatelier E.. (2013). Dietary intervention impact on gut microbial gene richness. Nature 500 (7464), 585–588. doi: 10.1038/nature12480 23985875

[B17] Crovesy L. Masterson D. Rosado E. L. (2020). Profile of the gut microbiota of adults with obesity: A systematic review. Eur. J. Clin. Nutr. 74 (9), 1251–1262. doi: 10.1038/s41430-020-0607-6 32231226

[B18] Doi Y. Kubo M. Yonemoto K. Ninomiya T. Iwase M. Tanizaki Y.. (2007). Liver enzymes as a predictor for incident diabetes in a Japanese population: The hisayama study. Obes. (Silver. Spring). 15 (7), 1841–1850. doi: 10.1038/oby.2007.218 17636103

[B19] Duan M. Wang Y. Zhang Q. Zou R. Guo M. Zheng H. (2021). Characteristics of gut microbiota in people with obesity. PloS One 16 (8), e255446. doi: 10.1371/journal.pone.0255446 PMC8354443 34375351

[B20] Engin A. (2017). The definition and prevalence of obesity and metabolic syndrome. Adv. Exp. Med. Biol. 960, 1–17. doi: 10.1007/978-3-319-48382-5_1 28585193

[B21] Fang C. Zhong H. Lin Y. Chen B. Han M. Ren H.. (2018). Assessment of the cPAS-based BGISEQ-500 platform for metagenomic sequencing. Gigascience 7 (3), 1–8. doi: 10.1093/gigascience/gix133 PMC5848809 29293960

[B22] Frank D. N. St A. A. Feldman R. A. Boedeker E. C. Harpaz N. Pace N. R. (2007). Molecular-phylogenetic characterization of microbial community imbalances in human inflammatory bowel diseases. Proc. Natl. Acad. Sci. U. S. A. 104 (34), 13780–13785. doi: 10.1073/pnas.0706625104 17699621 PMC1959459

[B23] Franzini M. Fornaciari I. Rong J. Larson M. G. Passino C. Emdin M.. (2013). Correlates and reference limits of plasma gamma-glutamyltransferase fractions from the framingham heart study. Clin. Chim. Acta 417, 19–25. doi: 10.1016/j.cca.2012.12.002 23247050 PMC4154585

[B24] Furusawa Y. Obata Y. Fukuda S. Endo T. A. Nakato G. Takahashi D.. (2013). Commensal microbe-derived butyrate induces the differentiation of colonic regulatory T cells. Nature 504 (7480), 446–450. doi: 10.1038/nature12721 24226770

[B25] Ganesan K. Chung S. K. Vanamala J. Xu B. (2018). Causal relationship between diet-induced gut microbiota changes and diabetes: A novel strategy to transplant faecalibacterium prausnitzii in preventing diabetes. Int. J. Mol. Sci. 19 (12), 3720. doi: 10.3390/ijms19123720 PMC6320976 30467295

[B26] Grundy S. M. Cleeman J. I. Daniels S. R. Donato K. A. Eckel R. H. Franklin B. A.. (2005). Diagnosis and management of the metabolic syndrome: An American heart Association/National heart, lung, and blood institute scientific statement. Circulation 112 (17), 2735–2752. doi: 10.1161/CIRCULATIONAHA.105.169404 16157765

[B27] Hiippala K. Kainulainen V. Kalliomaki M. Arkkila P. Satokari R. (2016). Mucosal prevalence and interactions with the epithelium indicate commensalism of sutterella spp. Front. Microbiol. 7. doi: 10.3389/fmicb.2016.01706 PMC5080374 27833600

[B28] Hsieh M. H. Ho C. K. Hou N. J. Hsieh M. Y. Lin W. Y. Yang J. F.. (2009). Abnormal liver function test results are related to metabolic syndrome and BMI in Taiwanese adults without chronic hepatitis b or c. Int. J. Obes. (Lond). 33 (11), 1309–1317. doi: 10.1038/ijo.2009.172 19752878

[B29] Huang G. Pan H. Zhu Z. Li Q. (2020). The complete genome sequence of bifidobacterium longum LTBL16, a potential probiotic strain from healthy centenarians with strong antioxidant activity. Genomics 112 (1), 769–773. doi: 10.1016/j.ygeno.2019.05.015 31226482

[B30] Kim H. R. Han M. A. (2018). Association between serum liver enzymes and metabolic syndrome in korean adults. Int. J. Environ. Res. Public Health 15 (8), 1658. doi: 10.3390/ijerph15081658 PMC6121325 30081587

[B31] Kostic A. D. Gevers D. Siljander H. Vatanen T. Hyotylainen T. Hamalainen A. M.. (2015). The dynamics of the human infant gut microbiome in development and in progression toward type 1 diabetes. Cell Host Microbe 17 (2), 260–273. doi: 10.1016/j.chom.2015.01.001 25662751 PMC4689191

[B32] Lan R. Wan Z. Xu Y. Wang Z. Fu S. Zhou Y.. (2021). Taurine reprograms mammary-gland metabolism and alleviates inflammation induced by streptococcus uberis in mice. Front. Immunol. 12. doi: 10.3389/fimmu.2021.696101 PMC8222520 34177964

[B33] Le Chatelier E. Nielsen T. Qin J. Prifti E. Hildebrand F. Falony G.. (2013). Richness of human gut microbiome correlates with metabolic markers. Nature 500 (7464), 541–546. doi: 10.1038/nature12506 23985870

[B34] Ley R. E. Turnbaugh P. J. Klein S. Gordon J. I. (2006). Microbial ecology: Human gut microbes associated with obesity. Nature 444 (7122), 1022–1023. doi: 10.1038/4441022a 17183309

[B35] Li F. Hullar M. A. Schwarz Y. Lampe J. W. (2009). Human gut bacterial communities are altered by addition of cruciferous vegetables to a controlled fruit- and vegetable-free diet. J. Nutr. 139 (9), 1685–1691. doi: 10.3945/jn.109.108191 19640972 PMC2728691

[B36] Li N. N. Li W. Feng J. X. Zhang W. W. Zhang R. Du S. H.. (2021). High alcohol-producing klebsiella pneumoniae causes fatty liver disease through 2,3-butanediol fermentation pathway *in vivo* . Gut. Microbes 13 (1), 1979883. doi: 10.1080/19490976.2021.1979883 34632939 PMC8510565

[B37] Li A. Li T. Gao X. Yan H. Chen J. Huang M.. (2021). Gut microbiome alterations in patients with thyroid nodules. Front. Cell Infect. Microbiol. 11. doi: 10.3389/fcimb.2021.643968 PMC8005713 33791245

[B38] Lindheim L. Bashir M. Munzker J. Trummer C. Zachhuber V. Leber B.. (2017). Alterations in gut microbiome composition and barrier function are associated with reproductive and metabolic defects in women with polycystic ovary syndrome (PCOS): A pilot study. PloS One 12 (1), e168390. doi: 10.1371/journal.pone.0168390 PMC5207627 28045919

[B39] Ling Z. Jin C. Xie T. Cheng Y. Li L. Wu N. (2016). Alterations in the fecal microbiota of patients with HIV-1 infection: An observational study in a chinese population. Sci. Rep. 6, 30673. doi: 10.1038/srep30673 27477587 PMC4967929

[B40] Liu Y. Jiang Q. Liu Z. Shen S. Ai J. Zhu Y.. (2021). Alteration of gut microbiota relates to metabolic disorders in primary aldosteronism patients. Front. Endocrinol. (Lausanne). 12. doi: 10.3389/fendo.2021.667951 PMC8415980 34484110

[B41] Lopez-Siles M. Duncan S. H. Garcia-Gil L. J. Martinezmedina M. (2017). Faecalibacterium prausnitzii: From microbiology to diagnostics and prognostics. ISME. J. 11 (4), 841–852. doi: 10.1038/ismej.2016.176 28045459 PMC5364359

[B42] Lun H. Yang W. Zhao S. Jiang M. Xu M. Liu F.. (2019). Altered gut microbiota and microbial biomarkers associated with chronic kidney disease. Microbiologyopen 8 (4), e678. doi: 10.1002/mbo3.678 PMC6460263 30088332

[B43] Ma X. Hua J. Li Z. (2008). Probiotics improve high fat diet-induced hepatic steatosis and insulin resistance by increasing hepatic NKT cells. J. Hepatol. 49 (5), 821–830. doi: 10.1016/j.jhep.2008.05.025 18674841 PMC2588670

[B44] Manco M. Putignani L. Bottazzo G. F. (2010). Gut microbiota, lipopolysaccharides, and innate immunity in the pathogenesis of obesity and cardiovascular risk. Endocr. Rev. 31 (6), 817–844. doi: 10.1210/er.2009-0030 20592272

[B45] Maya-Lucas O. Murugesan S. Nirmalkar K. Alcaraz L. D. Hoyo-Vadillo C. Pizano-Zarate M. L.. (2019). The gut microbiome of Mexican children affected by obesity. Anaerobe 55, 11–23. doi: 10.1016/j.anaerobe.2018.10.009 30366118

[B46] Mondo E. Barone M. Soverini M. D'Amico F. Cocchi M. Petrulli C.. (2020). Gut microbiome structure and adrenocortical activity in dogs with aggressive and phobic behavioral disorders. Heliyon 6 (1), e3311. doi: 10.1016/j.heliyon.2020.e03311 PMC6994854 32021942

[B47] Moratalla A. Gomez-Hurtado I. Moya-Perez A. Zapater P. Peiro G. Gonzalez-Navajas J. M.. (2016). Bifidobacterium pseudocatenulatum CECT7765 promotes a TLR2-dependent anti-inflammatory response in intestinal lymphocytes from mice with cirrhosis. Eur. J. Nutr. 55 (1), 197–206. doi: 10.1007/s00394-015-0837-x 25657013

[B48] Morrison D. J. Preston T. (2016). Formation of short chain fatty acids by the gut microbiota and their impact on human metabolism. Gut. Microbes 7 (3), 189–200. doi: 10.1080/19490976.2015.1134082 26963409 PMC4939913

[B49] Oh S. Y. Cho Y. K. Kang M. S. Yoo T. W. Park J. H. Kim H. J.. (2006). The association between increased alanine aminotransferase activity and metabolic factors in nonalcoholic fatty liver disease. Metabolism 55 (12), 1604–1609. doi: 10.1016/j.metabol.2006.07.021 17142131

[B50] Onat A. Can G. Ornek E. Cicek G. Ayhan E. Dogan Y. (2012). Serum gamma-glutamyltransferase: Independent predictor of risk of diabetes, hypertension, metabolic syndrome, and coronary disease. Obes. (Silver. Spring). 20 (4), 842–848. doi: 10.1038/oby.2011.136 21633402

[B51] Org E. Blum Y. Kasela S. Mehrabian M. Kuusisto J. Kangas A. J.. (2017). Relationships between gut microbiota, plasma metabolites, and metabolic syndrome traits in the METSIM cohort. Genome Biol. 18 (1), 70. doi: 10.1186/s13059-017-1194-2 28407784 PMC5390365

[B52] Pacifico L. Nobili V. Anania C. Verdecchia P. Chiesa C. (2011). Pediatric nonalcoholic fatty liver disease, metabolic syndrome and cardiovascular risk. World J. Gastroenterol. 17 (26), 3082–3091. doi: 10.3748/wjg.v17.i26.3082 21912450 PMC3158407

[B53] Palmas V. Pisanu S. Madau V. Casula E. Deledda A. Cusano R.. (2021). Gut microbiota markers associated with obesity and overweight in Italian adults. Sci. Rep. 11 (1), 5532. doi: 10.1038/s41598-021-84928-w 33750881 PMC7943584

[B54] Park E. Y. Lim M. K. Oh J. K. Cho H. Bae M. J. Yun E. H.. (2013). Independent and supra-additive effects of alcohol consumption, cigarette smoking, and metabolic syndrome on the elevation of serum liver enzyme levels. PloS One 8 (5), e63439. doi: 10.1371/journal.pone.0063439 23667618 PMC3646757

[B55] Qin J. Li Y. Cai Z. Li S. Zhu J. Zhang F.. (2012). A metagenome-wide association study of gut microbiota in type 2 diabetes. Nature 490 (7418), 55–60. doi: 10.1038/nature11450 23023125

[B56] Qin N. Yang F. Li A. Prifti E. Chen Y. Shao L.. (2014). Alterations of the human gut microbiome in liver cirrhosis. Nature 513 (7516), 59–64. doi: 10.1038/nature13568 25079328

[B57] Quevrain E. Maubert M. A. Michon C. Chain F. Marquant R. Tailhades J.. (2016). Identification of an anti-inflammatory protein from faecalibacterium prausnitzii, a commensal bacterium deficient in crohn's disease. Gut 65 (3), 415–425. doi: 10.1136/gutjnl-2014-307649 26045134 PMC5136800

[B58] Quintanilha M. F. Miranda V. C. Souza R. O. Gallotti B. Cruz C. Santos E. A.. (2022). Bifidobacterium longum subsp. longum 5(1A) attenuates intestinal injury against irinotecan-induced mucositis in mice. Life Sci. 289, 120243. doi: 10.1016/j.lfs.2021.120243 34922941

[B59] Requena T. Martinez-Cuesta M. C. Pelaez C. (2018). Diet and microbiota linked in health and disease. Food Funct. 9 (2), 688–704. doi: 10.1039/c7fo01820g 29410981

[B60] Sadik R. Bjornsson E. Simren M. (2010). The relationship between symptoms, body mass index, gastrointestinal transit and stool frequency in patients with irritable bowel syndrome. Eur. J. Gastroenterol. Hepatol. 22 (1), 102–108. doi: 10.1097/MEG.0b013e32832ffd9b 19701093

[B61] Saklayen M. G. (2018). The global epidemic of the metabolic syndrome. Curr. Hypertens. Rep. 20 (2), 12. doi: 10.1007/s11906-018-0812-z 29480368 PMC5866840

[B62] Sakon H. Nagai F. Morotomi M. Tanaka R. (2008). Sutterella parvirubra sp. nov. and megamonas funiformis sp. nov., isolated from human faeces. Int. J. Syst. Evol. Microbiol. 58 (Pt 4), 970–975. doi: 10.1099/ijs.0.65456-0 18398204

[B63] Schmitt F. Schneider M. Mathejczyk W. Weigand M. A. Figueiredo J. C. Li C. I.. (2021). Postoperative complications are associated with long-term changes in the gut microbiota following colorectal cancer surgery. Life (Basel). 11 (3), 246. doi: 10.3390/life11030246 33809741 PMC8002283

[B64] Shao L. Ling Z. Chen D. Liu Y. Yang F. Li L. (2018). Disorganized gut microbiome contributed to liver cirrhosis progression: A meta-Omics-Based study. Front. Microbiol. 9. doi: 10.3389/fmicb.2018.03166 PMC6315199 30631318

[B65] Sharpton S. R. Schnabl B. Knight R. Loomba R. (2021). Current concepts, opportunities, and challenges of gut microbiome-based personalized medicine in nonalcoholic fatty liver disease. Cell Metab. 33 (1), 21–32. doi: 10.1016/j.cmet.2020.11.010 33296678 PMC8414992

[B66] Sheng S. Chen J. Zhang Y. Qin Q. Li W. Yan S.. (2021). Structural and functional alterations of gut microbiota in males with hyperuricemia and high levels of liver enzymes. Front. Med. (Lausanne). 8. doi: 10.3389/fmed.2021.779994 PMC8640097 34869502

[B67] Shiraishi M. Tanaka M. Okada H. Hashimoto Y. Nakagawa S. Kumagai M.. (2019). Potential impact of the joint association of total bilirubin and gamma-glutamyltransferase with metabolic syndrome. Diabetol. Metab. Syndr. 11, 12. doi: 10.1186/s13098-019-0408-z 30740147 PMC6360758

[B68] Silva F. P. Inada A. C. Ribeiro F. M. Granja A. D. Freitas K. C. Avellaneda G. R.. (2018). An overview of novel dietary supplements and food ingredients in patients with metabolic syndrome and non-alcoholic fatty liver disease. Molecules 23 (4), 877. doi: 10.3390/molecules23040877 PMC6017470 29641459

[B69] Sokol H. Pigneur B. Watterlot L. Lakhdari O. Bermudez-Humaran L. G. Gratadoux J. J.. (2008). Faecalibacterium prausnitzii is an anti-inflammatory commensal bacterium identified by gut microbiota analysis of crohn disease patients. Proc. Natl. Acad. Sci. U. S. A. 105 (43), 16731–16736. doi: 10.1073/pnas.0804812105 18936492 PMC2575488

[B70] Song L. Huang Y. Liu G. Li X. Xiao Y. Liu C.. (2021). A novel immunobiotics bacteroides dorei ameliorates influenza virus infection in mice. Front. Immunol. 12. doi: 10.3389/fimmu.2021.828887 PMC8826429 35154087

[B71] Steinvil A. Shapira I. Ben-Bassat O. K. Cohen M. Vered Y. Berliner S.. (2010). The association of higher levels of within-normal-limits liver enzymes and the prevalence of the metabolic syndrome. Cardiovasc. Diabetol. 9, 30. doi: 10.1186/1475-2840-9-30 20633271 PMC2915953

[B72] Targher G. Byrne C. D. Tilg H. (2020). NAFLD and increased risk of cardiovascular disease: Clinical associations, pathophysiological mechanisms and pharmacological implications. Gut 69 (9), 1691–1705. doi: 10.1136/gutjnl-2020-320622 32321858

[B73] Truong D. T. Franzosa E. A. Tickle T. L. Scholz M. Weingart G. Pasolli E.. (2015). MetaPhlAn2 for enhanced metagenomic taxonomic profiling. Nat. Methods 12 (10), 902–903. doi: 10.1038/nmeth.3589 26418763

[B74] Wei Y. Li Y. Yan L. Sun C. Miao Q. Wang Q.. (2020). Alterations of gut microbiome in autoimmune hepatitis. Gut 69 (3), 569–577. doi: 10.1136/gutjnl-2018-317836 31201284

[B75] Yadav D. Lee M. Y. Kim J. Y. Ryu H. Huh J. H. Bae K. S.. (2017). Combined effect of initial and longitudinal increases in gamma-glutamyltransferase on incident metabolic syndrome: ARIRANG study. Yonsei. Med. J. 58 (4), 763–769. doi: 10.3349/ymj.2017.58.4.763 28540989 PMC5447107

[B76] Yin Y. N. Yu Q. F. Fu N. Liu X. W. Lu F. G. (2010). Effects of four bifidobacteria on obesity in high-fat diet induced rats. World J. Gastroenterol. 16 (27), 3394–3401. doi: 10.3748/wjg.v16.i27.3394 20632441 PMC2904885

[B77] Yoshida N. Emoto T. Yamashita T. Watanabe H. Hayashi T. Tabata T.. (2018). Bacteroides vulgatus and bacteroides dorei reduce gut microbial lipopolysaccharide production and inhibit atherosclerosis. Circulation 138 (22), 2486–2498. doi: 10.1161/CIRCULATIONAHA.118.033714 30571343

[B78] Yoshida N. Yamashita T. Osone T. Hosooka T. Shinohara M. Kitahama S.. (2021). Bacteroides spp. promotes branched-chain amino acid catabolism in brown fat and inhibits obesity. iScience 24 (11), 103342. doi: 10.1016/j.isci.2021.103342 34805797 PMC8586802

[B79] Yuan J. Chen C. Cui J. Lu J. Yan C. Wei X.. (2019). Fatty liver disease caused by high-Alcohol-Producing klebsiella pneumoniae. Cell Metab. 30 (4), 675–688. doi: 10.1016/j.cmet.2019.08.018 31543403

[B80] Yu J. Feng Q. Wong S. H. Zhang D. Liang Q. Y. Qin Y.. (2017). Metagenomic analysis of faecal microbiome as a tool towards targeted non-invasive biomarkers for colorectal cancer. Gut 66 (1), 70–78. doi: 10.1136/gutjnl-2015-309800 26408641

[B81] Yusof N. Hamid N. Ma Z. F. Lawenko R. M. Wan M. W. Collins D. A.. (2017). Exposure to environmental microbiota explains persistent abdominal pain and irritable bowel syndrome after a major flood. Gut. Pathog. 9, 75. doi: 10.1186/s13099-017-0224-7 29255490 PMC5729606

[B82] Zhang X. Mu Y. Yan W. Ba J. Li H. (2014). Alanine aminotransferase within reference range is associated with metabolic syndrome in middle-aged and elderly Chinese men and women. Int. J. Environ. Res. Public Health 11 (12), 12767–12776. doi: 10.3390/ijerph111212767 25513998 PMC4276645

[B83] Zhang X. Shen D. Fang Z. Jie Z. Qiu X. Zhang C.. (2013). Human gut microbiota changes reveal the progression of glucose intolerance. PloS One 8 (8), e71108. doi: 10.1371/journal.pone.0071108 24013136 PMC3754967

[B84] Zhou F. Zhou J. Wang W. Zhang X. J. Ji Y. X. Zhang P.. (2019). Unexpected rapid increase in the burden of NAFLD in China from 2008 to 2018: A systematic review and meta-analysis. Hepatology 70 (4), 1119–1133. doi: 10.1002/hep.30702 31070259

